# Inflammation as an exacerbation marker and target for prophylaxis against Coronavirus Disease 2019-related thrombosis

**DOI:** 10.7150/ijms.78911

**Published:** 2023-01-01

**Authors:** Daisuke Ono, Yuko Ohno, Yoshihiko Izumida, Hideaki Ohno, Hideaki Oka, Kyosuke Takeshita

**Affiliations:** 1 Department of Infectious Diseases and Infection Control, Saitama Medical Center, Saitama Medical University, Kawagoe, Saitama, Japan.; 2 Department of Clinical Laboratory, Saitama Medical Center, Saitama Medical University, Kawagoe, Saitama, Japan.; 3 Department of Endocrinology and Diabetes, Saitama Medical Center, Saitama Medical University, Kawagoe, Saitama, Japan.; 4 Department of General Internal Medicine, Saitama Medical Center, Saitama Medical University, Kawagoe, Saitama, Japan.

**Keywords:** COVID-19, SARS-CoV-2, inflammation, thrombosis, Immunosuppressants, liver transplant

## Abstract

**Objectives**: There are currently no appropriate markers and target for prophylaxis against COVID-19-related thrombosis, especially in the not-severe cases. We tested the hypothesis that inflammation is a suitable marker and target for prophylaxis against COVID-19-related thrombosis.

**Methods**: Data of all 32 COVID-19 patients admitted to Saitama Medical Center between January 1 and March 30, 2021, were analyzed. Patients were divided into severe (requiring oxygen, n=12) and non-severe (no requirement for oxygen, n=20), and also those with high C-reactive protein (CRP) level (cutoff value: 30 mg/L, n=21) and low-CRP (n=11). We also compared the clinical and laboratory data of a 46-year-old post-liver transplant male patient, who was treated with a combination of immunosuppressants (methylprednisolone, fludrocortisone, cyclosporine, and everolimus) with those of other COVID-19 patients, using the Smirnoff-Grubbs and Box plots tests.

**Results**: The levels of CRP, ferritin, lactate dehydrogenase, aspartate aminotransferase, and thrombin-antithrombin complex (TAT) were significantly higher in the high-severity group than the low-severity group; while other coagulation parameters were comparable. The time between onset of illness and blood levels of lactate dehydrogenase, fibrinogen, D-dimer, TAT, and plasmin alpha2-plasmin inhibitor complex (PIC) were significantly higher whereas lymphocyte count was significantly lower in the high-CRP group. Extremely low levels of TAT, PIC, and plasminogen activator inhibitor-1 (PAI-1) were recorded in the liver transplant patient treated with immunosuppressants. The TAT, PIC, and PAI-1 levels were deemed outliers.

**Conclusions**: Inflammation is a potentially suitable marker and target for prophylaxis against COVID-19-related thrombosis.

## Introduction

The prothrombotic tendency in severe acute respiratory syndrome coronavirus 2 (SARS-CoV-2) infection correlates with severe disease course. Prevention of thrombosis is an important aspect of the clinical management of coronavirus disease 2019 (COVID-19). However, there are currently no appropriate markers for the initiation of preventive anticoagulation, especially in the not-severe COVID-19 patients. Fibrinogen- or D-dimer-triggered anticoagulation does not seem to be clinically efficacious 1.

In addition to the lack of appropriate markers, there is no standardized protocol for thromboprophylaxis for the not-severe COVID-19 patients. In the ACTIV-4B trial, thromboprophylaxis with either aspirin or apixaban (prophylactic or therapeutic dose) did not elicit significant reduction in thrombosis, and at the same time adversely increased bleeding tendency 2. Thus, at present, clinical evidence suggests that prophylactic anticoagulation does not seem a proper therapeutic option.

The mechanism of COVID-19-related thrombosis is related to crosstalk among several inflammatory cascades and the thrombogenic properties of the host. Furthermore, thrombosis in such patients could rapidly lead to deterioration of the overall general condition. Previous studies in patients infected with COVID-19 suggested that suppression of immune dysregulation leads to inhibition of thrombus formation 3. Based on this point of view, various immunosuppressive agents are currently under consideration as the potential treatment of thrombosis.

Since the use of various anticoagulants has not been reported to be associated with favorable outcome, we analyzed changes in various indices of thrombosis in response to treatment with anti-inflammatory agents. In this study, we examined the hypothesis that inflammation could be an appropriate marker and target against COVID-19-related thrombosis. To test this hypothesis, we assessed the effects of a combination of immunosuppressants as prophylaxis against thrombosis.

## Methods

### COVID-19 patients

In addition to the post-liver transplant patient described below, we analyzed all 32 COVID-19 patients admitted to our hospital (Saitama Medical Center) between January 1 and March 30, 2021. Based on clinical assessment at the time of admission to the hospital, the patients were divided into two groups that describe the severity of COVID-19 infection; patients without (low-severity group) and those requiring oxygen therapy (high-severity group). The following parameters were compared at the time of admission; age, sex, severity (oxygen-demand, oxygen saturation <94%) and the National Institute of Allergy and Infectious Diseases (NIAID)-ordinal scale 4), comorbidities (hypertension, cardiovascular diseases, chronic kidney diseases, diabetes mellitus), and time between onset of illness and blood sampling. We also assessed several other variables related to severity, including characteristics of systemic inflammation and coagulation at the time of admission: C-reactive protein (CRP), procalcitonin, ferritin, lactate dehydrogenase, aspartate aminotransferase, alanine aminotransferase, serum creatinine, lymphocyte count, prothrombin time-international normalized ratio (PT-INR), activated partial thromboplastin time (APTT), and the levels of fibrinogen, antithrombin III, D-dimer, thrombin-antithrombin complex (TAT), plasmin alpha2-plasmin inhibitor complex (PIC), and plasminogen activator inhibitor-1 (PAI-1) (Table 1). Blood sampling were performed at the time of hospital admission. For the serum sample preparation, blood collection tubes were centrifuged at 2270 g after letting them stand for fifteen minutes. For the plasma sample, blood tubes with 3.2% sodium citrate (the ratio of sodium citrate and blood was one to nine) were thoroughly mixed and centrifuged at 2000 g for ten minutes. For the blood count and fraction sample, blood tubes with ethylenediaminetetraacetic acid were used for the analysis. Each analysis was conducted with a XN-9000 (Sysmex Co., Kobe, Japan) for blood count, a Labospect 008 alpha® (Hitachi High-Tech Co., Tokyo, Japan) for biochemical markers, a Roche COBAS 8000 device (Roche Diagnostic, Basel, Switzerland) for procalcitonin, a CP-3000 (Sekisui Medical, Tokyo, Japan) for coagulation markers, and a STASIA (LSI Medience Co., Tokyo, Japan) for PAI-1, PIC, and TAT, respectively.

### Comparison based on C-reactive protein level

To examine the pathological role of inflammation as a potential risk factor in COVID-19-related thrombotic tendency, patients were divided into two groups based on the level of CRP (using a cutoff value of 30 mg/L, similar to the study of Cheng et al. 5). The role of inflammation was assessed by comparing the same variables mentioned above in the two CRP groups.

### Case report of post-liver transplant COVID-19-positive patient

The patient was a 46-year-old man who had undergone living-donor liver transplantation at our hospital for non-compensatory alcoholic cirrhosis two years before current admission. The medical history included hypertension and chronic renal failure, and he was being treated with immunosuppressants to prevent transplant rejection, including methylprednisolone (8 mg/day), fludrocortisone (0.05 mg/day), cyclosporine (100 mg/day), and everolimus (1.5 mg/day). On admission with COVID-19 infection, the vital signs were normal, including oxygenation. However, a CT scan of the chest showed several fresh glass-ground shadows in the peripheries of both lungs. The patient was not considered suitable for anticoagulation therapy due to the poor renal function. We decided against the use of additional immunosuppressants, based on the post-transplant status and lack of demand for oxygen. After admission, the clinical course was uneventful. He was discharged on the 10^th^ day after admission without any severe complications.

### Statistical analysis

Comparisons between two groups were conducted using the χ^2^-test, Student's t-test, and Mann-Whitney U-test, as appropriate. Data were analyzed using Prism 7 software (GraphPad Inc., San Diego, CA). A two-tailed p-value <0.05 was considered statistically significant. For data of the post-liver transplant patient, we examined whether the values (log10) of on-admission blood tests were significant outliers compared to other COVID-19 patients, using the Smirnoff-Grubbs test (α=0.05) and Box plots test (turkey method interquartile range).

## Results

### Differences between COVID-19 low- and high-severity groups

The patients were divided in two groups according to the severity of infection, as reflected by the need for oxygen therapy. There were no differences in several clinical characteristics, such as age, sex, comorbidities, and time between illness onset and blood sampling between the two groups. The levels of various makers of inflammation (e.g., CRP and ferritin), and those of organ injury (e.g., lactate dehydrogenase, aspartate aminotransferase) were significantly higher in the high-severity group (n=12, p=0.04, 0.01, 0.006, and 0.05, respectively, Table 1). With regard to the coagulation profile, TAT levels were higher in the high-severity group than the low-severity group (n=20, p=0.009), but other coagulation indices were comparable (Table 1).

### Differences between COVID-19 low- and high-CRP groups

There were no differences between the low- and high-CRP groups with regard to age, sex, and various comorbidities. The time between the onset of illness and blood sampling and levels of lactate dehydrogenase, fibrinogen, D-dimer, TAT, and PIC were significantly higher, and lymphocyte count was significantly lower in the high-CRP group compared with the low-CRP group (p=0.03, <0.001, 0.006, and 0.05, respectively) (Table 2).

### Reduced thrombotic tendency in the post-liver transplant patient

The tendency for thrombosis was extremely low in the post-liver transplant patient, who was being treated with combination of oral immunosuppressants. Blood tests conducted on admission showed extremely low levels of TAT (<0.10 ng/mL), PIC (<0.05 pg/mL), and PAI-1 (<0.30 ng/mL) (Table 2). The results of other coagulation tests included PT-INR 0.83, APTT 33.4 sec, D-dimer 1.08 µg/mL, fibrinogen 650 mg/dL, and antithrombin III 192%. Comparison of the data of this patient to those of other COVID-19 patients using both Smirnoff-Grubbs test and Box plots test indicated the blood TAT, PIC and PAI-1 levels of the patient were outliers.

## Discussion

In this study, we demonstrated a causal relationship between CRP, an inflammatory marker, and COVID-19-related prothrombotic state. We also showed that the combined use of various immunosuppressants could suppress inflammation-induced thrombosis.

Immune abnormalities and subsequent thrombosis are the main underlying pathogenic mechanisms of severe COVID-19 infection 3. When the SARS-CoV-2 virus invades the alveoli, immune cells, including monocytes and macrophages, secrete excessive amounts of inflammatory cytokines, causing a cytokine storm 3. Inflammatory cytokines, such as IL-6, IL-1, IL-10, TNF-α, are released, and monocyte-derived tissue factor, PAI-1 expression, fibrin, neutrophil extracellular traps, and platelets cause thrombosis not only in microvessels but also in macrovessels 3. In particular, it is known that the release of IL-6 contributes to the activation of coagulation through differentiation of Th17 cells, leading to severe COVID-19 infection 6. When SARS-CoV-2 infection caused an “inflammatory storm”, not only associated cytokines rise sharply, but some inflammatory biomarkers also increase, such as CRP, serum ferritin, and procalcitonin 7. Reportedly, levels of CRP, serum ferritin, and procalcitonin were significantly increased according to severity of COVID-19, and increases in these inflammatory biomarkers indicate a high risk of severe illness and poor prognosis 7. In this study, we defined CRP as the hyperinflammatory biomarkers because IL-6 directly increases serum CRP levels and plays critical roles in COVID-19-rerated multiorgan failure 8.

In this study, we compared the data of our COVID-19 cohort by disease severity, and the results showed comparable coagulation indices except for higher TAT levels in the high-severity group. In contrast, comparison based on inflammation marker CRP showed significantly higher levels of several coagulation indices, such as fibrinogen, D-dimer, TAT, and PIC in the high-CRP group, compared to the low-CRP group. These results suggest that inflammation seems a better marker of worsening COVID-19 than severity. The recent study shows that the suboptimal fibrinolytic response in COVID-19 patients is directly attributable to elevated levels of PAI-1 9, but the results in this study did not reach statistical significance (P value = 0.09). The small sample size may attribute to a lack of power. In addition, as PAI-1 is rapidly synthesized by inflammatory cytokines 10, insignificant delay in blood sampling in the high severity group could blunt the peak of PAI-1 levels.

If inflammation is an appropriate marker for COVID-19-related thrombosis, it can also potentially serve as an appropriate target for prophylaxis against COVID-19-related thrombosis. Indeed, in the post-liver transplant patient treated with several immunosuppressants (steroids, cyclosporine, and everolimus), the patient had low levels of inflammatory marker CRP in blood tests conducted on admission, with notably markedly low levels of coagulation parameters. Among the coagulation parameters, the levels of TAT, PIC, and PAI-1 were low compared with other conventional coagulation values. In general, TAT indirectly reflects thrombin production (thrombus formation), PIC suggests activation of the fibrinolytic system, and an increase in PAI-1, an inhibitor of fibrinolysis, indicates a general tendency toward thrombus formation. We speculate that in our patient, the three immunosuppressants acted alone or synergistically to suppress the inflammatory response caused by COVID-19 infection and also suppressed the subsequent coagulation abnormalities that could lead to COVID-19 deterioration.

What about the specific immunosuppressants, steroids, cyclosporine, and everolimus, taken by the post-liver transplant patient? The mechanism of action of each of these immunosuppressants is different and unique. Steroids are currently the most effective immunosuppressive agents used for COVID-19 infection, with the highest level of evidence for their efficacy 11. Steroids are recommended only for severe patients who require oxygenation (no recommendation for patients without need for oxygen), although they have been suggested to potentially induce thrombosis 12. Cyclosporine works by inhibiting the aggregation of T cells 13. The mammalian target of rapamycin (mTOR) inhibitor works on PAI-1 by acting on IL-6 and the JAK/STAT signaling pathway to inhibit hypercoagulation 4 14. In particular, the mTOR inhibitor, everolimus, could be worthy of attention because the mechanism of mTOR inhibitor against COVID-19 infection has not been thoroughly investigated, and it could be one of the effective therapeutic agents. Interestingly, a previous report suggested that mTOR inhibitors could have favorable biological effects on SARS-CoV-2 15. Considering the above studies, it is possible that each immunosuppressive agent, acted in a complementary manner in our patient, to suppress the high inflammatory state caused by COVID-19 infection and prevented hypercoagulation at the same time.

Because the present study has a single-center retrospective cross-sectional design, limitations include a small sample number and patient selection bias. This should be considered when interpreting the results. Further large-scale studies are needed to confirm the study findings on the relationship between CRP and COVID-19-related prothrombotic state.

## Conclusions

In our cohort of 32 COVID-19 patients, COVID-19 severity was related to organ injury rather than to prothrombotic tendency. Furthermore, the tendency for thrombosis was more evident in patients with high CRP levels. Inflammation is a potentially suitable marker for thrombotic status in COVID-19, and administration of a combination of immunosuppressants could perhaps provide prophylaxis against COVID-19-related thrombosis.

## Figures and Tables

**Table 1:** Clinical characteristics of patients with high- and low-severity coronavirus disease-2019 (COVID-19) infection

	Low-severity group (n=20)		High-severity group (n=12)		P value
	Average	Median	Average	Median	
Age	65	69	71	73	0.28
Sex, male (%)	14 (70.0)		9 (75.0)		>0.99
**Comorbidities, at least one, number (%)**					
Hypertension	8 (40.0)		7 (58.3)		0.25
Cardiovascular disease	4 (20.0)		3 (25.0)		0.66
Chronic kidney disease	2 (10.0)		2 (16.7)		>0.99
Diabetes mellitus	6 (30.0)		2 (16.7)		0.40
Time between onset of illness and blood sampling (days)	7	6	9	9	0.37
**Variables of severity factors (including Inflammatory variables)**					
C-reactive protein (mg/L) (Normal range, <0.14)	50.5	26.0	103	89.0	0.04*
Procalcitonin (ng/mL) (Normal range, <0.5)	0.85	0.10	0.39	0.20	0.25
Ferritin (ng/mL) (Normal range, 18-440)	434	296	1230	973	0.01**
Lactate dehydrogenase (units/L) (Normal range, 124-222)	286	293	419	418	0.006**
Aspartate transaminase (units /L) (Normal range, 13-30)	38.2	30.5	61.1	45.5	0.05 *
Alanine transaminase (units/L) (Normal range, 10-42)	32.0	27.5	50.4	27.5	0.67
Serum Creatinine (mg/dL) (Normal range, 0.65-1.07)	1.89	0.90	1.87	0.96	0.60
Lymphocyte count ×103/µL	15.9	15.5	10.2	10.3	0.07
**Variables of coagulation system**					
Prothrombin time-international normalized ratio (Normal range, 0.90-1.10)	1.01	1.00	1.04	1.01	0.88
Activated partial thromboplastin time (second) (Normal range, 24.0-39.0)	36.0	35.8	38.2	37.7	0.47
Fibrinogen (mg/dL) (Normal range, 200-400)	537	496	653	632	0.14
Antithrombin III (%) (Normal range, 70-130)	92.5	91.5	96.0	92.0	0.88
D-dimer (µg/mL) (Normal range, <1.0)	1.47	1.10	1.46	1.31	0.57
Thrombin-antithrombin complex (ng/mL) (Normal range, <3)	2.69	1.90	26.5	4.12	0.009**
Plasmin alfa2-plasmin inhibitor complex (pg/mL) (Normal range, <0.8)	1.60	1.26	2.60	2.29	0.06
Plasminogen activator inhibitor-1 (ng/mL) (Normal range, <50)	50.8	32.2	46.8	48.5	0.26

Data are average, median, or number (percentage) of patients. P value for differences between the low- and high-severity groups. *P<0.05, **P<0.01, by Mann-Whitney U test.

**Table 2:** Clinical characteristics of patients of the low- and high-CRP groups

	Liver transplant patient	Low-CRP group (n=11)		High-CRP group (n=21)		P value
		Average	Median	Average	Median	
Age	46	63	64	70	71	0.45
Sex, male (%)		7 (63.6)		16 (76.2)		0.70
**Comorbidities, at least one, number (%)**						
Hypertension	-	4 (36.4)		11 (52.4)		0.47
Cardiovascular disease	-	2 (18.2)		6 (28.6)		0.68
Chronic kidney disease	-	3 (27.3)		4 (19.0)		0.67
Diabetes mellitus	-	2 (18.2)		2 (9.5)		0.59
Time between onset of illness and blood sampling (days)	3	6	5	8	8	0.03*
**Variables of severity factors (including Inflammatory variables)**						
C-reactive protein (mg/L) (Normal range, <0.14)	7	11.3	9.7	101	88.6	<0.001***
Procalcitonin (ng/mL) (Normal range, <0.5)	0.20	0.15	0.10	0.95	0.20	0.10
Ferritin (ng/mL) (Normal range, 18-440)	1940	458	277	876	571	0.15
Lactate dehydrogenase (units/L) (Normal range, 124-222)	341	257	244	377	403	0.005**
Aspartate transaminase (units /L) (Normal range, 13-30)	68.0	38.9	32.0	50.9	41.0	0.54
Alanine transaminase (units/L) (Normal range, 10-42)	105	35.4	28.0	40.8	26.0	0.81
Serum Creatinine (mg/dL) (Normal range, 0.65-1.07)	2.28	2.79	0.91	1.82	0.94	0.49
Lymphocyte count ×103/µL	9.00	19.9	19.8	10.6	11.5	<0.001***
**Variables of coagulation system**						
Prothrombin time-international normalized ratio (Normal range, 0.90-1.10)	0.83	0.96	0.94	1.05	1.02	0.08
Activated partial thromboplastin time (second) (Normal range, 24.0-39.0)	33.4	36.3	36.2	37.1	36.7	0.55
Fibrinogen (mg/dL) (Normal range, 200-400)	650	480	496	633	626	0.03*
Antithrombin III (%) (Normal range, 70-130)	192	94.2	91.0	93.7	92.0	0.88
D-dimer (µg/mL) (Normal range, <1.0)	1.08	1.06	0.71	1.68	1.31	0.009**
Thrombin-antithrombin complex (ng/mL) (Normal range, <3)	0.1^†^	1.84	1.75	16.8	3.80	0.002**
Plasmin alfa2-plasmin inhibitor complex (pg/mL) (Normal range, <0.8)	0.05^†^	1.20	1.24	2.36	2.25	0.04*
Plasminogen activator inhibitor-1 (ng/mL) (Normal range, <50)	0.3^†^	30.8	31.7	59.0	40.2	0.09

Data are average, median, or number (percentage) of patients. A CRP cutoff value of 30 mg/L measured at the time of admission was used, as described in the text. Data of the single patient with liver transplantation treated with steroids, cyclosporine A, and everolimus are included in the Table. Smirnoff-Grubbs test (α=0·05) and Box plots test (turkey method, interquartile range) were used to compare data of this patient findings against those of the other COVID-19 patients. † Variables deemed to be outliers. *P<0.05, **P<0.01, ***P<0.001, comparison between the low- and high-CRP groups, by Mann-Whitney U test.
